# Percepción de los estudiantes del uso de los simuladores en la práctica clínica odontológica en universidades peruanas

**DOI:** 10.21142/2523-2754-1402-2026-282

**Published:** 2026-04-04

**Authors:** Jhon Paul Iakov Mezarina-Mendoza, Enna Lucila Garavito-Chang, Elizabeth Chávez-Sánchez, Mitze Gaby Ramírez-Chaupis, Carlos Michell Gálvez Ramírez, Sara Castañeda-Sarmiento

**Affiliations:** 1 Facultad de Odontología, Universidad Nacional Mayor de San Marcos. Lima, Perú. jmezarinam@unmsm.edu.pe, cgalvezr@unmsm.edu.pe, scastanedas@unmsm.edu.pe Universidad Nacional Mayor de San Marcos Facultad de Odontología Universidad Nacional Mayor de San Marcos Lima Peru jmezarinam@unmsm.edu.pe cgalvezr@unmsm.edu.pe scastanedas@unmsm.edu.pe; 2 Universidad Norbert Wiener. Lima, Perú. enna.garavito@uwiener.edu.pe Universidad Privada Norbert Wiener Universidad Norbert Wiener Lima Peru enna.garavito@uwiener.edu.pe; 3 Universidad Tecnológica de los Andes. Abancay, Perú. echavezs@utea.edu.pe Universidad Tecnológica de los Andes Universidad Tecnológica de los Andes Abancay Peru echavezs@utea.edu.pe; 4 Escuela Profesional de Odontología, Universidad Nacional Hermilio Valdizán. Huánuco, Perú. mitzeramirezc@gmail.com Universidad Nacional Hermilio Valdizán Escuela Profesional de Odontología Universidad Nacional Hermilio Valdizán Huánuco Peru mitzeramirezc@gmail.com

**Keywords:** entrenamiento simulado, educación en odontología, simulación de paciente, percepción estudiantil, simulation training, dental education, patient simulation, student perception

## Abstract

**Introducción:**

La simulación en odontología permite recrear entornos clínicos controlados que favorecen el desarrollo de competencias en un contexto seguro. Aunque la evidencia internacional resalta sus beneficios, los estudios siguen siendo limitados en el Perú.

**Metodología:**

Se realizó un estudio descriptivo y transversal con 222 estudiantes de odontología de cinco universidades peruanas. Se aplicó un cuestionario validado (V de Aiken = 0,906; Alfa de Cronbach = 0,946) que evaluó utilidad didáctica, desarrollo de competencias y experiencia en simulación.

**Resultados:**

El 79,1% de los participantes consideró que los simuladores facilitan la aplicación de conocimientos teóricos; el 72,9% reportó mejoras en habilidades visuales y motoras, y el 68,8% indicó mayor confianza clínica.

**Conclusiones:**

Los simuladores constituyen herramientas valiosas en la educación odontológica al fortalecer habilidades técnicas y confianza antes de la práctica clínica en pacientes reales. Su impacto podría optimizarse mediante una mejor supervisión y materiales de mayor calidad.

## Abstract (en)

**Introduction:**

Simulation in dentistry provides controlled environments that replicate clinical situations, allowing students to acquire competencies safely. Although international evidence highlights its benefits, studies in Peru remain scarce.

**Methods:**

A cross-sectional descriptive study was conducted with 222 dental students from five Peruvian universities. A validated questionnaire (Aiken’s V=0.906; Cronbach’s α=0.946) assessed didactic utility, competency development, and simulation experience.

**Results:**

Most students considered simulators useful for applying theoretical knowledge (79.1%), improving visual and motor skills (72.9%), and increasing clinical confidence (68.8%).

**Conclusions:**

Simulators are valuable tools in dental education, strengthening skills and confidence before clinical practice. Their effectiveness could be enhanced by improving supervision and material quality.

## INTRODUCCIÓN

La simulación en odontología se refiere al uso de entornos artificiales o herramientas diseñadas para imitar escenarios clínicos reales, lo que permite a los estudiantes entrenar, practicar y perfeccionar sus habilidades en un ambiente seguro y controlado. Este enfoque ha demostrado ser altamente eficaz para favorecer la adquisición de competencias clínicas, potenciar el desarrollo del pensamiento crítico y garantizar un aprendizaje seguro que disminuya los riesgos para los pacientes, con lo cual se consolida como una estrategia pedagógica fundamental en la educación en ciencias de la salud ^1^.

El desarrollo tecnológico ha dado lugar a una amplia variedad de simuladores, que abarcan desde modelos tradicionales, como los fantomas y *typodonts*, hasta simuladores virtuales como *Simodont* y *PerioSim*, además de plataformas robóticas que incorporan realidad aumentada y retroalimentación háptica. Estas innovaciones han contribuido a superar limitaciones propias de los métodos convencionales de enseñanza, tales como la escasez de piezas dentarias naturales y la variabilidad inherente a la práctica clínica con pacientes ^2^^,^^3^.

La evidencia internacional respalda que el uso de simuladores favorece el desarrollo de la destreza manual, la coordinación motriz visual y la confianza clínica de los estudiantes, al mismo tiempo que promueve la adopción de hábitos ergonómicos y prácticas de bioseguridad ^4^^-^^6^ De igual manera, su implementación se ha relacionado con una transición más fluida desde la formación preclínica hacia la atención de pacientes reales, lo que refuerza la relevancia de su incorporación en el currículo de las carreras de salud ^7^^,^^8^.

En Latinoamérica se han documentado avances en la implementación de simuladores; sin embargo, la producción científica con alto nivel de rigurosidad aún se mantiene en un nivel moderado ^9^. En el caso de Perú, si bien existen iniciativas institucionales y publicaciones que reportan experiencias locales ^10^^-^^12^, el número de estudios indexados en bases como SciELO, PubMed y Scopus que evalúen resultados educativos mediante diseños metodológicos robustos continúa siendo limitado. Esta carencia refuerza la necesidad de generar estudios multicéntricos que evalúen la percepción estudiantil sobre la simulación clínica en odontología.

## MATERIALES Y MÉTODOS

El estudio de tipo observacional, descriptivo y transversal fue aprobado por el comité de ética de la Facultad de Odontología de la Universidad Nacional Mayor de San Marcos, con código de aprobación N.° 053-CEI-FO-2024.

### Validación del instrumento

Se utilizó un cuestionario diseñado y validado por Mezarina-Mendoza *et al*. ^13^. La validez de contenido se confirmó mediante el coeficiente V de Aiken (0,906). La confiabilidad se evaluó con la prueba de alfa de Cronbach, en la que se obtuvo un valor de 0,946, lo que evidencia alta consistencia interna. El cuestionario incluyó tres dimensiones: a) instrumento didáctico, b) aspectos favorecidos y c) competencias y evaluación.

La primera sección recopiló datos generales (sexo, ciclo de estudios y experiencia en simuladores), mientras que la segunda consistió en 23 ítems valorados en una escala de Likert de cinco categorías desde “muy de acuerdo” hasta “muy en desacuerdo”.

### Muestra

El tamaño de muestra calculado con Epidat 4.2 fue de 205, utilizando un 95% de confianza, 5% de margen de error y una proporción del 76%. La muestra final estuvo compuesta por 222 estudiantes, lo que superó el mínimo requerido y aseguró la validez y solidez estadística del estudio. La proporción esperada fue igual al 76%, considerada de estudio previo realizado por Fernández-Sagredo *et al*. ^12^. Para los criterios de inclusión fueron considerados estudiantes mayores de 18 años con matrícula vigente, mientras que se excluyeron cuestionarios incompletos, estudiantes de intercambio y estudiantes de primero y segundo año.

### Recolección y discriminación de data

Para la recolección de la data se desarrolló y distribuyó una encuesta en línea utilizando Microsoft Forms, del 6 de mayo al 28 de junio de 2024, y el cuestionario fue enviado por correo electrónico. Las facultades o escuelas de odontología participantes fueron la Universidad Nacional Mayor de San Marcos (UNMSM), la Universidad Nacional Tecnológica de los Andes (UTEA), la Universidad de Huánuco (UDH), la Universidad Hermilio Valdizán (UNHEVAL) y la Universidad Norbert Wiener (UWIENER). Las cinco universidades participantes desarrollan actualmente sus actividades académicas conforme a sus directivas institucionales y planes de estudio. En este contexto, emplean simuladores odontológicos (fantomas), los cuales presentan características similares en todas las instituciones.

Los datos recopilados fueron exportados y organizados en una base de datos diseñada en una hoja de cálculo de Microsoft Excel® (Microsoft Corporation, Redmond, EE. UU.). Esta base de datos consolidó la información sobre las características demográficas de los estudiantes universitarios de odontología en Perú, así como las respuestas obtenidas de los ítems de la escala de Likert del cuestionario.

### Análisis estadístico 

El análisis de los datos se llevó a cabo utilizando el *software* estadístico IBM™ SPSS Statistics (International Business Machines Corporation, Armonk, Nueva York, EE. UU.). Se realizó un análisis estadístico descriptivo para resumir y presentar las características demográficas de la muestra, como edad, sexo, año de estudio y universidad de procedencia. Los resultados se presentaron en tablas que detallan el número de casos (n.º) y el porcentaje (%) para cada categoría. Para la variable edad, también se calcularon la media y la desviación estándar (DE).

La prueba de Kruskal-Wallis (K-W) se aplicó para determinar si existían diferencias estadísticamente significativas en la percepción de los simuladores dentales en función de las características demográficas de los estudiantes. Los resultados de esta prueba se reportaron con el valor del estadístico K-W y el valor de probabilidad (p) menor a 0,05 (p < 0,05) sugiere que existe una diferencia estadísticamente significativa 

## RESULTADOS

### Características demográficas de los estudiantes 

La mayoría de los estudiantes encuestados se encontraba entre 20 y 24 años (59,9%), con una media de 24,7 años, lo que corresponde a la población que ya está en etapas avanzadas de su formación. Predominaron las mujeres (67,1%), lo cual refleja el incremento de estas en las facultades del país. En cuanto al año académico, sobresalen los estudiantes de internado (36%) y cuarto año (35,1%). La mayor proporción provino de la Universidad Tecnológica de los Andes (30,2%), seguida por la Universidad Norbert Wiener (27,4%) y la UNMSM (20,2%) (Tabla 1).

**Tabla 1:** Características demográficas de estudiantes universitarios de escuelas de odontología del Perú, 2024

Característica		N.º	%
Edad (años)	< 20	2	0,9
	20-24	133	59,9
	25-29	54	24,3
	30-más	33	14,9
	Media ± DE	24,7 ± 3,7		
Sexo	Masculino	73	32,9
	Femenino	149	67,1
Año de estudio	3.^o^	12	5,4
	4.^o^	78	35,1
	5.^o^	52	23,4
	Internado	80	36,0
Universidad	U. de Huánuco	12	5,4
	U. Hermilio Valdizán	37	16,6
	UNMSM	45	20,2
	U. Norbert Wiener	61	27,4
	U. Tecnológica de los Andes	67	30,2
Total		222	100,0

### Percepción de los simuladores como instrumento clínico 

Los estudiantes valoraron positivamente a los simuladores como recurso de aprendizaje. Un 78,8% afirmó que facilitan la aplicación de conocimientos teóricos y un 69,4% destacó su utilidad para identificar aciertos y errores en la práctica. Además, un 66,6% consideró que los fantomas aportan realismo a las actividades. Sin embargo, hubo opiniones divididas con respecto a la calidad de los dientes artificiales y la necesidad de supervisión constante, lo que sugiere limitaciones en la implementación de estos recursos (Tabla 2).

**Tabla 2:** Respuestas con Likert a ítems del cuestionario sobre uso de simuladores en la práctica clínica odontológica en la dimensión “instrumento clínico”, de estudiantes en universidades peruanas, 2024

Ítems	Respuestas según Likert										Total		
	Muy de acuerdo		De acuerdo		Neutral		Desacuerdo		Muy en desacuerdo			
	N.º	%	N.º	%	N.º	%	N.º	%	N.º	%	N.º	%
1. Permite la aplicación de conocimientos teóricos.	65	29,3	110	49,5	33	14,9	10	4,5	4	1,8	222	100,0
2. Permite al alumno detectar aciertos y errores.	57	25,7	97	43,7	49	22,1	15	6,8	4	1,8	222	100,0
3. No necesita supervisión permanente a diferencia de prácticas en pacientes reales.	33	14,9	68	30,6	65	29,3	44	19,8	12	5,4	222	100,0
4. Los tipos de dientes utilizados son adecuados para las prácticas de los distintos cursos.	34	15,3	79	35,6	71	32,0	28	12,6	10	4,5	222	100,0
5. La práctica se hace más realista al usar fantomas.	48	21,6	100	45,0	62	27,9	9	4,1	3	1,4	222	100,0
6., El tema a desarrollar y el avance es el mismo en toda la clase.	43	19,4	103	46,4	54	24,3	18	8,1	4	1,8	222	100,0
7. Permite efectivizar tiempos en procedimientos	55	24,8	113	50,9	42	18,9	8	3,6	4	1,8	222	100,0
8. El uso de simuladores ayuda a adquirir destrezas manuales más rápido que con los métodos tradicionales.	49	22,1	105	47,3	49	22,1	15	6,8	4	1,8	222	100,0
Total	384	21,6	775	43,6	425	23,9	147	8,3	45	2,5	1776	100,0

### Aspectos favorecidos por el uso de simuladores 

Los simuladores son altamente valorados por su capacidad para prevenir la contaminación cruzada y fomentar el uso de barreras de protección tanto para el operador como para el paciente; un 67,2% de los estudiantes estuvo de acuerdo o muy de acuerdo con esta afirmación. Además, el 74,2% coincidió en que los simuladores contribuyen al desarrollo de hábitos ergonómicos durante las prácticas. Un 67,5% de los encuestados también indicó que los simuladores promueven el uso de medidas de protección. Sin embargo, el 44,1% mostró una postura neutral en cuanto a la percepción de ahorro económico que ofrecen los simuladores. Por otro lado, el 68,8 % de los estudiantes manifestó un incremento en su confianza clínica tras el uso de simuladores, lo que evidencia su impacto positivo en el proceso formativo y en la preparación para la práctica profesional (Tabla 3).

**Tabla 3:** Respuestas con Likert a ítems del cuestionario sobre uso de simuladores en la práctica clínica odontológica en la dimensión “aspectos favorecidos”, de estudiantes en universidades peruanas, 2024

Ítems	Respuestas según Likert										Total		
	Muy de acuerdo		De acuerdo		Neutral		Desacuerdo		Muy en desacuerdo			
	N.º	%	N.º	%	N.º	%	N.º	%	N.º	%	N.º	%
9. El uso de simuladores evita la contaminación cruzada.	52	23,4	97	43,7	54	24,3	15	6,8	4	1,8	222	100,0
10. La práctica en simuladores crea el hábito del uso de barreras de protección para operador y paciente.	49	22,1	101	45,5	50	22,5	18	8,1	4	1,8	222	100,0
11. La distribución de los simuladores en el área de práctica es adecuada para la práctica de posiciones operador-paciente de manera ergonómica.	47	21,2	117	52,7	45	20,3	10	4,5	3	1,4	222	100,0
12. La práctica en simuladores desarrolla el hábito de trabajar ergonómicamente.	50	22,5	116	52,3	45	20,3	8	3,6	3	1,4	222	100,0
13. La práctica en simuladores incrementa la confianza de los alumnos al tener que realizar procedimientos en pacientes reales.	54	24,3	99	44,6	50	22,5	16	7,5	3	1,4	222	100,0
14. La práctica en el uso de simuladores reduce el riesgo de cometer errores en pacientes en reales.	41	18,5	64	28,8	77	34,7	27	12,2	13	5,9	222	100,0
15. El uso adecuado de los simuladores permitirá el ahorro de los estudiantes.	30	13,5	77	34,7	98	44,1	13	5,9	4	1,8	222	100,0
16. El material de las piezas dentarias usadas en el simulador no produce un efecto medioambiental negativo.	51	23,0	118	53,2	45	20,3	6	2,7	2	0,9	222	100,0
Total	374	21,1	789	44,4	464	26,1	113	6,4	36	2,0	1776	100,0

### Desarrollo de competencias mediante simuladores 

En el ámbito de la formación avanzada, el uso de simuladores en odontología se ha consolidado como una herramienta esencial para el desarrollo de competencias prácticas. En este sentido, el 72,9% de los estudiantes reconoce que estas tecnologías contribuyen de manera significativa a mejorar sus habilidades visuales y motoras. Asimismo, el 67,6% señala que los simuladores fortalecen tanto las destrezas clínicas como preclínicas, lo que favorece una preparación integral. De igual forma, el 61,8% de los encuestados refiere que el empleo de simuladores facilita una mayor precisión en la selección de técnicas, lo cual resalta su valor en la toma de decisiones y en la construcción de criterios clínicos más sólidos (Tabla 4).

**Tabla 4:** Respuestas con Likert a ítems del cuestionario sobre uso de simuladores en la práctica clínica odontológica en la dimensión “competencias”, de estudiantes en universidades peruanas, 2024

Ítems	Respuestas según Likert										Total	
	Muy de acuerdo		De acuerdo		Neutral		Desacuerdo		Muy en desacuerdo			
	N.º	%	N.º	%	N.º	%	N.º	%	N.º	%	N.º	%
17. Permite mejora de habilidades visuales y motoras.	47	21,2	115	51,8	49	22,1	8	3,6	3	1,4	222	100,0
18. Permite mejorar habilidades clínicas y preclínicas	43	19,4	107	48,2	57	25,7	11	5,0	4	1,8	222	100,0
19. El nivel de realismo visual y táctil permite la adecuada manipulación del instrumental al realizar procedimientos clínicos	35	15,8	91	41,0	68	30,6	23	10,4	5	2,3	222	100,0
20. Permite seleccionar técnicas adecuadas para el caso presentado	41	18,5	96	43,2	68	30,6	14	6,3	3	1,4	222	100,0
21. Al haber errado en la fantoma el alumno tiene la posibilidad de solucionar el problema y aprender del mismo	54	24,3	110	49,5	49	22,1	7	3,2	2	0,9	222	100,0
Total	220	19,8	519	46,7	291	26,2	63	5,7	17	1,5	1110	100,0

### Evaluación a través de simuladores 

En la evaluación de habilidades odontológicas mediante simuladores, el 64,9% de los estudiantes valoró positivamente el uso de matrices de evaluación, al reconocer su utilidad para unificar criterios de calificación. Del mismo modo, el mismo porcentaje destacó la presentación de casos idénticos, lo que favorece una evaluación más objetiva. No obstante, un 27,7% adoptó una postura neutral, evidencia de que no todos perciben plenamente las ventajas que ofrece la estandarización de evaluaciones a través de simuladores (Tabla 5).

**Tabla 5:** Respuestas con Likert a ítems del cuestionario sobre uso de simuladores en la práctica clínica odontológica en la dimensión “evaluación”, de estudiantes en universidades peruanas, 2024.

Ítems	Respuestas según Likert										Total		
	Muy de acuerdo		De acuerdo		Neutral		Desacuerdo		Muy en desacuerdo			
	N.º	%	N.º	%	N.º	%	N.º	%	N.º	%	N.º	%
22. Se utiliza matriz de evaluación para unificar criterios al calificar la práctica del estudiante en el simulador.	43	19,4	101	45,5	65	29,3	10	4,5	3	1,4	222	100,0
23. Facilita la evaluación al presentar casos idénticos a solucionar	37	16,7	107	48,2	62	27,9	10	4,5	6	2,7	222	100,0
Total	80	18,0	208	46,8	127	28,6	20	4,5	9	2,0	444	100,0

### Influencia de las características demográficas en la percepción de los simuladores 

El análisis estadístico no evidenció diferencias significativas en la percepción de los simuladores según edad (p = 0,119) o sexo (p = 0,107). No obstante, sí se encontraron diferencias entre universidades (p = 0,001): los estudiantes de la UNMSM y la Universidad Tecnológica de los Andes mostraron actitudes más favorables hacia el uso de simuladores. Esto resalta el peso del contexto institucional en la forma en que los estudiantes valoran estas herramientas (Tabla 6).

**Tabla 6:** Puntaje medio en respuestas con Likert a ítems del cuestionario sobre uso de simuladores en la práctica clínica odontológica según edad sexo y universidad de estudiantes en universidades peruanas

Características	Indicador del puntaje			Prueba K-W
	Media	DE	Mediana	
Edad				
< 20	3,83	0,06	3,83	K-W = 5,86
20-24	3,84	0,60	3,80	p = 0,119
25-29	3,57	0,71	3,57	
30-+	3,71	0,68	3,74	
Sexo				
Masculino	3,66	0,70	3,70	K-W = 2,59
Femenino	3,80	0,62	3,80	p = 0,107
Universidad				
U. de Huánuco	3,50	0,61	3,50	K-W = 19,64
U. Hermilio Valdizán	3,29	0,80	3,48	p = 0,001
UNMSM	3,91	0,51	3,87	
U. Norbert Wiener	3,84	0,60	3,78	
U. Tecnológica de los Andes	3,87	0,59	3,83	
Total	3,76	0,65	3,78	

p > 0,05 no existe relación estadística significativa.

p < 0,01 existe relación estadística altamente significativa.

## DISCUSIÓN 

Los resultados obtenidos evidencian una percepción mayoritariamente favorable hacia los simuladores odontológicos como instrumentos clínicos. La mayoría de los estudiantes reconoció que facilitan la aplicación de conocimientos teóricos (79,1%) y la identificación de aciertos y errores (69,4%), lo que coincide con investigaciones previas que destacan el valor de la simulación como recurso pedagógico para trasladar la teoría al ámbito práctico ^1^^,^^4^^,^^7^^,^^8^. Sin embargo, la ambivalencia observada en torno a aspectos como necesidad de supervisión, la calidad de los dientes artificiales empleados e incluso la naturalidad del tacto al tejido, y la de las máscaras faciales de los fantomas refleja limitaciones aún presentes en el realismo de los simuladores, los cuales son descritos en la literatura ^9^^,^^11^^,^^15^. Estos hallazgos sugieren que, además de la infraestructura tecnológica, es indispensable una adecuada integración curricular y un acompañamiento docente que optimice la experiencia formativa ^11^^,^^15^^,^^19^^,^^20^.

En cuanto a los aspectos favorecidos, los estudiantes valoraron positivamente la contribución de los simuladores a la bioseguridad, particularmente en la prevención de la contaminación cruzada (67,2%). Este hallazgo es consistente con estudios que resaltan el papel de la simulación en la adopción temprana de prácticas seguras, tanto para los estudiantes como para los pacientes. ^3^^,^^5^^,^^10^^,^^18^. De igual modo, el 74,2% de los participantes destacó su aporte en el fomento de hábitos ergonómicos ^16^, mientras que el 68,8% señaló un incremento en la confianza clínica, lo que refuerza su utilidad en la reducción de la ansiedad previa a la atención de pacientes reales ^6^^,^^9^^,^^18^.

En relación con el desarrollo de competencias, el 72,9% de los encuestados reportó mejoras en habilidades visuales y motoras, y un 61,8% reconoció una mayor precisión en la selección de técnicas. Estos resultados se alinean con la evidencia internacional que respalda el rol de la simulación en el fortalecimiento de la toma de decisiones clínicas y en la construcción de criterios sustentados en evidencia ^4^^,^^8^^,^^11^. Asimismo, corroboran la validez del instrumento de medición empleado, previamente desarrollado y validado en el contexto peruano ^13^.

Respecto a la evaluación, el 64,9% valoró el uso de matrices de calificación y la presentación de casos idénticos, lo que coincide con la literatura que enfatiza la estandarización como una ventaja en la objetividad del proceso evaluativo ^11^. Sin embargo, un 28,7% adoptó una postura neutral, lo que sugiere cierta resistencia o falta de confianza en la sustitución de la evaluación clínica tradicional por la simulación. Esto coincide con lo señalado por Baltera *et al*. ^9^, quienes advierten que la aceptación de nuevas metodologías educativas requiere procesos de adaptación progresiva. Este hallazgo es consistente con estudios que resaltan el papel de la simulación en la adopción temprana de prácticas seguras, tanto para los estudiantes como para los pacientes ^3^^,^^5^^,^^10^^,^^17^^,^^18^. De igual modo, el 74,2% de los participantes destacó su aporte en el fomento de hábitos ergonómicos ^16^, mientras que el 68,8% señaló un incremento en la confianza clínica, lo que refuerza su utilidad en la reducción de la ansiedad previa a la atención de pacientes reales ^6^^,^^9^^,^^18^.

En relación con el desarrollo de competencias, el 72,9% de los encuestados reportó mejoras en habilidades visuales y motoras, y un 61,8% reconoció una mayor precisión en la selección de técnicas. Estos resultados se alinean con la evidencia internacional que respalda el rol de la simulación en el fortalecimiento de la toma de decisiones clínicas y en la construcción de criterios sustentados en evidencia ^4^^,^^8^^,^^11^. Asimismo, corroboran la validez del instrumento de medición empleado, previamente desarrollado y validado en el contexto peruano ^13^.

Un hallazgo relevante fue la ausencia de diferencias significativas en la percepción del uso de simuladores según edad o sexo, lo que coincide con investigaciones previas que señalan que las características sociodemográficas no condicionan de manera sustancial la valoración de la simulación ^11^. En contraste, se identificaron diferencias entre universidades (p = 0,001), y fueron más favorables las percepciones en la UNMSM y en la Universidad Tecnológica de los Andes. Este patrón refuerza la importancia del contexto institucional, en el que factores como la disponibilidad de recursos, la capacitación docente y la integración curricular determinan en gran medida la efectividad de la simulación en el aprendizaje ^10^^,^^17^^,^^19^^,^^20^.

Por otro lado, la neutralidad reportada respecto al ahorro económico (44,1%) indica que los estudiantes aún no perciben un beneficio tangible en términos de costos. Este resultado debe interpretarse con cautela, considerando que la adquisición, mantenimiento y actualización de simuladores generan gastos elevados que, en algunos casos, pueden trasladarse a la matrícula o limitar su disponibilidad en instituciones con menos recursos ^9^.

En conjunto, los resultados confirman que los simuladores constituyen una herramienta valiosa en la educación odontológica peruana, pero también evidencian desafíos relacionados con el realismo, la supervisión docente, la percepción de costos y la aceptación de la evaluación estandarizada. Estos aspectos deben ser considerados en el diseño de políticas educativas que impulsen la innovación curricular y aseguren una implementación equitativa y sostenible de la simulación clínica en el país.

Un hallazgo cualitativamente relevante se refiere a la posible influencia de la experiencia clínica en la percepción de los estudiantes. En nuestro estudio, los alumnos de años superiores (quinto año e internado), quienes ya tienen experiencia directa con pacientes, pueden valorar los simuladores desde una perspectiva más contextualizada, basada en la transición del entorno simulado al clínico. Si bien el análisis estadístico no mostró diferencias significativas en las puntuaciones medias globales entre los años de estudio (p > 0,05), es plausible que la valoración de los estudiantes de internado esté matizada por su experiencia práctica. Por ejemplo, podrían apreciar con mayor profundidad cómo la simulación previa mitigó la ansiedad y redujo el riesgo de errores en sus primeras atenciones reales, un beneficio ampliamente respaldado por la literatura ^6^^,^^9^^,^^18^. Esta apreciación, aunque no se refleje en una diferencia estadística en una escala de Likert, subraya el papel fundamental de la simulación como un puente seguro y efectivo hacia la práctica clínica, lo que consolida su valor en la formación de competencias que son validadas posteriormente en el escenario real ^8^^,^^11^.

Este estudio presenta limitaciones vinculadas a la heterogeneidad en la disponibilidad y nivel de implementación de ambientes de simulación entre las universidades participantes, lo que pudo influir en la percepción de los estudiantes. Asimismo, el empleo de un diseño transversal y un muestreo no probabilístico restringe la posibilidad de extrapolar los resultados a contextos más amplios. Finalmente, la neutralidad observada frente a beneficios económicos y a la estandarización de evaluaciones refleja retos de adaptación y aceptación que deberán abordarse en futuros estudios comparativos y longitudinales.

Se recomienda fortalecer la integración de los simuladores en el currículo odontológico, para asegurar su alineación con objetivos pedagógicos específicos y el desarrollo de competencias críticas. La implementación de modelos híbridos que combinen simulación con tutorías personalizadas podría optimizar la retroalimentación y el acompañamiento estudiantil. Del mismo modo, resulta esencial evaluar de manera periódica el realismo y la funcionalidad de los simuladores, así como analizar su costo-beneficio para garantizar su sostenibilidad. Finalmente, se sugiere promover investigaciones comparativas entre instituciones, con el fin de identificar buenas prácticas que orienten políticas educativas hacia una adopción más efectiva de la simulación en la formación clínica.

## CONCLUSIÓN

La presente investigación evaluó la percepción de los estudiantes sobre el uso de simuladores en la práctica clínica odontológica en cinco universidades peruanas. Los resultados confirman que los simuladores se consolidan como herramientas valiosas en la formación, al facilitar el desarrollo de competencias técnicas y fortalecer la preparación previa a la atención de pacientes. Pese a las limitaciones identificadas, la simulación mostró un impacto positivo en el aprendizaje, ya que incrementa la confianza y las habilidades clínicas de los estudiantes. La optimización de la supervisión docente y la mejora en la calidad de los materiales podrían potenciar aún más su efectividad, lo que aseguraría una transición más sólida hacia escenarios clínicos reales y contribuiría a la formación integral de futuros odontólogos.

## Financiamiento:

El financiamiento de la presente investigación fue asumido íntegramente por los autores, sin apoyo económico de instituciones públicas, privadas o sin fines de lucro
